# Identifying a miRNA signature for predicting the stage of breast cancer

**DOI:** 10.1038/s41598-018-34604-3

**Published:** 2018-10-31

**Authors:** Srinivasulu Yerukala Sathipati, Shinn-Ying Ho

**Affiliations:** 1 Institute of Bioinformatics and Systems Biology, National Chiao Tung University, Hsinchu, Taiwan; 2 Department of Biological Science and Technology, National Chiao Tung University, Hsinchu, Taiwan; 3 Center For Intelligent Drug Systems and Smart Bio-devices (IDS2B), National Chiao Tung University, Hsinchu, Taiwan

## Abstract

Breast cancer is a heterogeneous disease and one of the most common cancers among women. Recently, microRNAs (miRNAs) have been used as biomarkers due to their effective role in cancer diagnosis. This study proposes a support vector machine (SVM)-based classifier SVM-BRC to categorize patients with breast cancer into early and advanced stages. SVM-BRC uses an optimal feature selection method, inheritable bi-objective combinatorial genetic algorithm, to identify a miRNA signature which is a small set of informative miRNAs while maximizing prediction accuracy. MiRNA expression profiles of a 386-patient cohort of breast cancer were retrieved from The Cancer Genome Atlas. SVM-BRC identified 34 of 503 miRNAs as a signature and achieved a 10-fold cross-validation mean accuracy, sensitivity, specificity, and Matthews correlation coefficient of 80.38%, 0.79, 0.81, and 0.60, respectively. Functional enrichment of the 10 highest ranked miRNAs was analysed in terms of Kyoto Encyclopedia of Genes and Genomes and Gene Ontology annotations. Kaplan-Meier survival analysis of the highest ranked miRNAs revealed that four miRNAs, hsa-miR-503, hsa-miR-1307, hsa-miR-212 and hsa-miR-592, were significantly associated with the prognosis of patients with breast cancer.

## Introduction

Breast cancer is one of the major leading causes of death among women, and it accounts for 14% of cancer deaths worldwide^1,2^. There are different types of breast carcinomas depending on the specific cells in the breast that are affected; most breast cancers are a type of adenocarcinoma. According the American Joint Committee on Cancer, the three features used to stage breast cancer are the size of the primary breast tumour (T), the spread of cancer to lymph nodes (N) and distant metastasis (M)^3^. In the TNM staging system, the T category represents the primary breast tumour and the spread within the tumour. The T category comprises stages T1 to T4 based on the tumour size. T1 tumours are subdivided into T1a, T1b and T1c, and the tumour size is >10 mm and ≤2 cm in dimension. T2 tumours are >2 cm, T3 tumours are >5 cm, and T4 tumours are any size and may spread to the breast skin or chest wall^3^. The estimated numbers of invasive and *in situ* breast cancer cases and breast cancer deaths in 2013 in the United States are 32,340, 64,640 and 39,620, respectively^4^. Approximately 252,710 new cases and 40,610 breast cancer deaths are estimated for US women in 2017 according to the surveillance, epidemiology, and end result programme (SEER 2017) statistics. Breast cancer survival rates are associated with the stage of the cancer. The 5-year survival rates for stages I, II and III are 100%, 93%, and 72%, respectively; unfortunately, the 5-year survival rate for stage IV breast cancer is only 22%^5^. Despite the advances in the treatment of breast cancer, metastatic breast cancer remains incurable, and mortality rate is still high due to the emergence of therapy-resistant cancer cells^6^ and limitations in the current treatment strategies. A better understanding of the molecular markers that affect breast tumours at different stages may lead to the development of new therapeutic strategies.

Recent evidence demonstrated that molecular marker-based targeted therapies have potential for the prognosis and diagnosis of various diseases. Molecular target-based studies focused on advances in microRNA (miRNA) expression profiling because of their prominent role in tumour development and metastasis. MiRNAs are small noncoding RNAs that regulate gene expression and are involved in human carcinogenesis^7^. Over the past few years, many studies reported the significant role of miRNAs in the molecular pathogenesis of breast tumours. MiRNA profiling studies have identified miRNAs that are aberrantly expressed in breast tumours and their functions. For instance, miRNAs such as miR-125b, miR-145, miR-155, and miR-21 are significantly deregulated in breast tumour tissues compared to normal tissue^8^. Potential association between miRNA and breast neoplasm has been predicted in studies^9,10^. Functionally, miRNAs are act as tumour suppressor^11^ and oncogene^12^ in breast tumour progression and metastasis. Gene expression and miRNA expression profiling has been used to classify different tumour types^13,14^. However, it has been confirmed that miRNA expression profiles can classify tumour types more accurately than gene expression profiles^14^.

Machine learning methods have been developed for cancer survival calculation, risk classification and prognosis prediction in various cancers, including breast cancer^15–17^. Several researchers have used different machine learning models and the Wisconsin breast cancer dataset to categorize benign and malignant breast cancers. For instance, M.F. Akay has used a support vector machine (SVM) combined with feature selection for a medical decision making system to diagnose breast cancer^18^. Abonyi and Szeifert have used a supervised rule-based fuzzy classifier to categorize benign and malignant breast cancers^19^. Pena-Reyes and Sipper have utilized a fuzzy-genetic algorithm method to classify benign and malignant breast tumours^20^. In addition, other well-known machine learning methods, such as the feed forward neural network algorithm^21^, the C4.5 decision tree method^22^, the linear discreet analysis method^23^ and the neuron-fuzzy technique^24^, have been developed for breast cancer diagnosis. Most machine learning methods developed for breast cancer classification using breast tumor images^25^ and gene/miRNA expression profiles^26^ to distinguish molecular subtypes^27^. Shimomura *et al*. identified five miRNAs to distinguish the breast cancer from other cancer types^28^. However, there are few studies of identifying the miRNA signature associated with the breast cancer stage for exploring the molecular level changes at various breast cancer stages.

Although there are methodologies for breast cancer treatment, challenges regarding early stage detection of breast tumours exist. Early stage detection may help to obtain a better treatment diagnosis. Therefore, we explored whether miRNA expression profiling could be used to categorize early stage breast tumours accurately. In this study, we collected the breast cancer data from the cancer genome atlas (TCGA) database and proposed a SVM-based classifier called SVM-BRC to categorize early stage and advanced stage patients with breast cancer using their miRNA expression profiles. SVM-BRC is based on an SVM incorporating an optimal feature selection method referred to as the inheritable bi-objective combinatorial genetic algorithm (IBCGA)^29^. We retrieved the miRNA expression profile data on 386 patients with breast cancer, with 193 patients in the early stage and the remaining 193 patients at an advanced stage groups. To the best of our knowledge, this is the first study to use miRNA expression profiles to identify the miRNA signature for predicting the breast cancer stage. SVM-BRC identified a signature consisting of 34 of 503 miRNAs that can distinguish early stage breast cancer patients from advanced stage breast cancer patients and achieved a 10-fold cross-validation (10-CV) mean accuracy, sensitivity, specificity, and Matthews correlation coefficient (MCC) of 80.38%, 0.79, 0.81, and 0.60, respectively. Further, we ranked the identified miRNAs based on the MED scores. The 10 highest ranked miRNAs were analysed based on their involvement in breast cancer and other cancer types. Functional enrichment of the 10 highest ranked miRNAs were analysed using Kyoto Encyclopedia of Genes and Genomes (KEGG) and Gene Ontology (GO) annotations. Kaplan-Meier survival analysis of the identified miRNAs revealed that four miRNAs among the 10 highest ranked miRNAs, hsa-miR-503, hsa-miR-1307, hsa-miR-212 and hsa-miR-592, were significantly associated with the overall survival of patients with breast cancer.

## Results and Discussion

### Prediction performance of SVM-BRC

**Figure 1:**
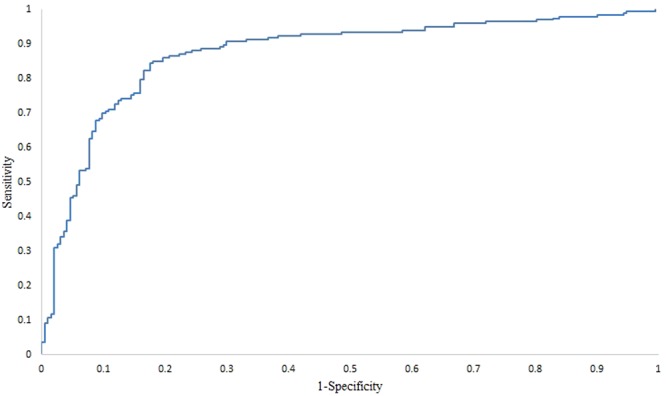
SVM-BRC performance evaluation using the ROC curve. The area under the ROC curve is 0.87 using a 386-patient breast cancer cohort.

We used a dataset consisting of 386 patients with breast cancer and 503 miRNA expression profiles. The dataset was divided into early stage (Stages I & II) and advanced stage (Stages III & IV) groups. Then, we attempted to categorize the early stage and the advanced stage groups using miRNA expression alone. The proposed SVM-BRC includes the feature selection algorithm IBCGA to select a significant miRNA signature that is associated with the tumour stage of breast cancer patients. SVM-BRC identified a miRNA signature (34 miRNAs) that can classify early stage and advanced stage groups and achieved a 10-CV mean accuracy, sensitivity, specificity, and MCC of 80.38% ± 1.55%, 0.79 ± 2.7, 0.81 ± 2.26, and 0.60 ± 0.03, respectively. SVM-BRC achieved a 10-CV accuracy, sensitivity, specificity, MCC and AUC of 83.16%, 0.84, 0.81, 0.66 and 0.87, respectively (shown in Table 1), and a jackknife test accuracy of 63.89%. The prediction performance of SVM-BRC was evaluated using a receiver operating curve (ROC), as shown in Fig. 1.

**Table 1:** Comparison of SVM-BRC with the some classifiers for the 386-patient breast cancer cohort.

Method	10-CV accuracy (%)	Sensitivity	Specificity	MCC
SVM-BRC-Mean	80.38 ± 1.55	0.79 ± 2.7	0.81 ± 2.26	0.60 ± 0.03
SVM-BRC-Best	83.16	0.84	0.81	0.66
Random forest	66.83	0.66	0.67	0.33
Multilayer perceptron	57.25	0.57	0.57	0.14
SMO	62.69	0.62	0.63	0.25
Naïve Bayes	64.50	0.63	0.65	0.29
Decision tree	50.25	0.50	0.50	0.01

We compared SVM-BRC with some machine learning methods of Weka such as Random forest (RF), Multilayer perceptron (MLP), Sequential minimal optimization (SMO), Naïve Bayes, and Decision tree. We used information gain for feature selection and Ranker attribute evaluator method, and obtained 14 miRNAs to distinguish early stage and advanced stage groups. The accuracies of RF, MLP, SMO, Naïve Bayes, and Decision tree methods using the 14 miRNAs with 10-CV were 66.83%, 57.25%, 62.69%, 64.50%, and 50.25% respectively. The results of performance comparison are shown in Table 1. The performance of SVM-BRC is much better than the other machine learning methods in distinguish the early stage and advanced stage groups.

### Prioritizing the miRNA signature

We ranked the miRNAs identified by SVM-BRC using main effect difference (MED) analysis^30^. The 10 highest ranked miRNAs based on their contribution to the prediction accuracy are hsa-miR-200c, hsa-miR-503, hsa-miR-1307, hsa-miR-361, hsa-miR-212, hsa-miR-592, hsa-miR-1185-1, hsa-miR-146b, hsa-miR-1468, and hsa-miR-769. The 10 highest ranked miRNAs and their MED scores are listed in Table 2. The 34 miRNA signature and their rankings are shown in Supplementary Table 1. Further, the significance of the 10 highest ranked miRNAs in breast cancer is discussed.

**Table 2:** Ten highest ranked miRNAs and feature knockout analysis of individual miRNAs.

Rank	miRNA	MED scores	Accuracy difference (%)
1	hsa-miR-200c	69.68	20.99
2	hsa-miR-503	65.02	20.73
3	hsa-miR-1307	48.44	21.25
4	hsa-miR-361	47.92	21.25
5	hsa-miR-212	46.89	20.99
6	hsa-miR-592	46.89	19.95
7	hsa-miR-1185-1	43.26	20.73
8	hsa-miR-146b	43.26	19.69
9	hsa-miR-1468	34.45	21.25
10	hsa-miR-769	30.82	20.47

#### Hsa-miR-200c

Hsa-miR-200c scored 69.68 and ranked one according to the MED ranking index, which means that the contribution of this miRNA is higher than that of the others. The miR-200 family of miRNAs possesses a unique role in cancer stem cells^31^, neurogenesis^32^, and chemosensitivity^33^. Hsa-miR-200c is aberrantly expressed in several cancers, including breast cancer. A retrospective analysis of 210 breast tumour samples revealed that hsa-miR-200c expression was associated with poor distant relapse-free survival^34^. A luciferase reporter assay study reported that hsa-miR-200c regulates cancer stem cell functions such as proliferation and self-renewal; miR-200c modulates the expression of the BM1 protein, which is an essential stem cell self-renewal regulator in breast cancer stem cells^35^. It is also observed that hsa-miR-200c suppresses the tumourigenicity of breast cancer stem cells^35^. This miRNA targets class III beta tubulin and increases the chemosensitivity in breast tumours^33^. Hsa-miR-200c is also significantly expressed in several other tumours, such as bladder cancer^36^, colorectal cancer^37^ and ovarian cancer^38^.

#### Hsa-miR-503

Hsa-miR-503 expression was found to be downregulated in breast cancer cells, and overexpression of this miRNA reduced cell proliferation by targeting CCND1^39^. A quantitative RT-PCR study involving screening a series of 12 inflammatory breast cancer cells showed that hsa-miR-503 was differently expressed and was used as a predictor for an inflammatory breast cancer phenotype^40^. Recently, overexpression of hsa-miR-503 was found in breast cancer tissue and plasma compared to that in healthy tissue; upregulation of this miRNA in breast cancer cells suppresses the expression of the epithelial-mesenchymal transition-related protein SMAD2 and the epithelial marker protein E-cadherin^41^. Experimental evidence showed that hsa-miR-503 regulates the oncogene ZNF217 and that higher expression of this miRNA is associated with improved survival in breast cancer^42^. Hsa-miR-503 acts as a tumour suppressor by targeting DDHD2 in breast cancer cells^43^.

#### Hsa-miR-1307

Hsa-miR-1307 was found to be upregulated in breast cancer. Hsa-miR-1307 was differentially expressed with a fold-change of 0.36 between breast cancer and the adjacent normal control tissue^44^. Hsa-miR-1307 expression was upregulated in BRCA1-associated breast carcinoma compared to that in the normal counterparts^45^.

#### Hsa-miR-361

A miRNA expression profiling study of 376 human miRNAs reported that hsa-miR-361 expression was downregulated in MCF-7 docetaxel-resistant breast cancer cells^46^. A screening study of miRNAs related to different subtypes of breast cancers showed that hsa-miR-361 was upregulated in metastatic breast tumours^47^. A microarray-based study of 375 breast tumour cases revealed that overexpression of hsa-miR-361 is correlated with the better disease-free survival in patients with breast cancer^48^. Downregulation of hsa-miR-361 was observed in 60 breast cancer tissues; hsa-miR-361 targets FGFR1 and MMP-1, resulting in inhibition of glycolysis and invasion in breast cancer cells^49^.

#### Hsa-miR-212

A case study of patients diagnosed with breast invasive ductal carcinoma reported that hsa-miR-212 was significantly downregulated in breast tumours by 0.328-fold and that this reduced expression was prominent in high grade breast tumours^50^. Hsa-miR-212 expression was downregulated in 30 paired triple-negative breast cancer samples, and its expression inhibited cell migration and invasion during cancer progression by targeting Prrx2^51^.

#### Hsa-miR-592

A real-time PCR study of a nonmetastatic breast cancer cell line reported the overexpression of hsa-miR-592^52^. Antonio Colaprico *et al*. identified differentially expressed miRNA-regulating pathway crosstalk between breast cancer and healthy samples; hsa-miR-592 expression was approximately twenty-three times higher in breast cancer samples than in healthy samples and regulated the extrinsic prothrombin activation pathway^53^. Recently, marked downregulation of hsa-miR-592 was observed in a breast cancer cell line compared to that in a normal breast cell line and further, hsa-miR-592 acted as tumour suppressor by targeting the transforming growth factor β-2 in breast cancer^54^.

#### Hsa-miR-146b

Hsa-miR-146b was downregulated and negatively regulated nuclear factor-kappaB, resulting in a reduction of the metastatic potential in breast cancer cells^55^. Higher expression of hsa-miR-146b induced interleukin-6 expression and signal transducer and activator transcription 3 phosphorylation, and this expression was positively correlated with survival in some breast cancer subtypes^56^. Hsa-miR-146a and hsa-miR-146b were found to be the most expressed in breast cancer metastasis suppressor 1-expressing cells, and upregulation of hsa-miR-146b was observed in the MDA-MB-435 breast cancer cell line^57^. A reporter assay study of triple negative breast tumours reported that hsa-miR-146b negatively regulates BRCA1 in triple negative sporadic breast cancer^58^. An RT-PCR study of 120 young women with primary breast tumours and 130 patients with breast fibroadenoma reported that downregulation of hsa-miR-146b expression in breast cancer cells was associated with the development and deterioration of breast cancer^59^.

#### Hsa-miR-769

Examination using the Nanostring nCounter assay on 43 miRNAs reported that hsa-miR-769 can inhibit the expression of N-myc downstream-regulated gene 1 upon reoxygenation in the breast adenocarcinoma cell line MCF-7 and that overexpression of hsa-miR-769 significantly enhanced apoptosis^60^. A study of triple negative breast cancer comparing African-American and non-Hispanic white women reported that 26 miRNAs, including hsa-miR-769, were differentially expressed between these groups^61^. Hsa-miR-769 found to be upregulated with a log2-fold change of 1.355 between triple negative breast cancer in African-American and non-Hispanic white women^61^. Differential expression of hsa-miR-769 was also found in male breast cancers^62,63^.

Our analysis of the 10 highest ranked miRNAs acknowledged that two miRNAs, hsa-miR-1185-1 and hsa-miR-1468, among the 10 highest ranked miRNAs are not directly involved in breast cancer but are implicated in other cancers. For instance, hsa-miR-1185-1 expression was abnormally low in Alzheimer’s disease^64^ and atherosclerosis^65^. The expression of hsa-miR-1468 was upregulated in hepatocellular carcinoma tissue^66^. Dysregulation of hsa-miR-1468 was observed in epithelial ovarian cancer^67^. Hsa-miR-1468 was significantly associated with the recurrence-free survival in lung adenocarcinoma^68^. Therefore, these two miRNAs are important molecules to validate further in breast cancer. Eight miRNAs among the 10 highest ranked miRNAs are involved not only in breast cancer but also in several major cancer types.

**Figure 2:**
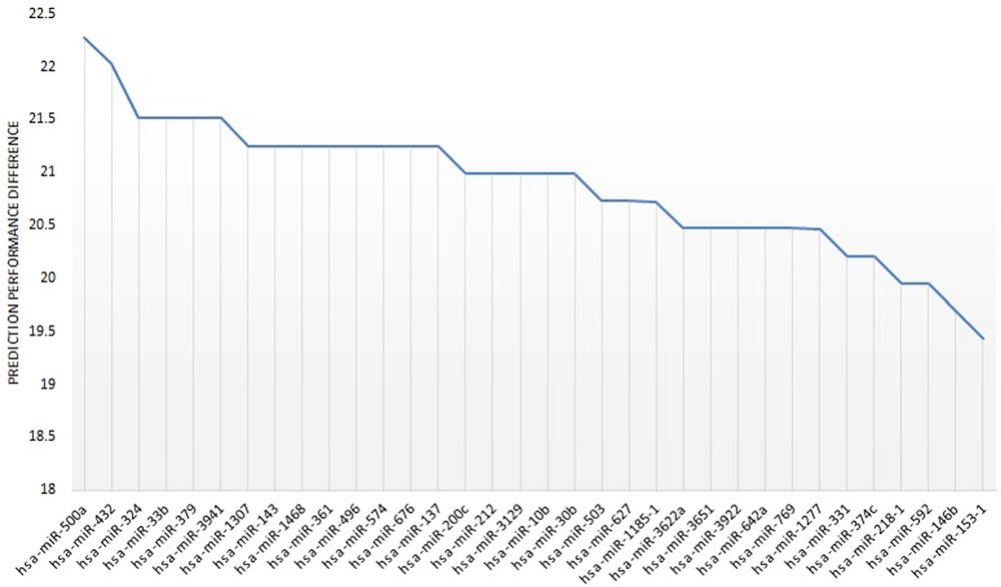
Feature knockout analysis. Prediction performance difference for individual miRNAs using feature knockout analysis.

Additionally, we employed miRNA knockout analysis to observe the difference in the prediction performance by removing one miRNA from the signature. Each miRNA of the 10 highest ranked miRNAs can affect the prediction performance with a mean accuracy difference of 20.73 ± 0.54. We report the results of knockout of the 10 highest ranked miRNAs in Table 2. The accuracy difference after removing each miRNA is depicted in Fig. 2. The accuracy differences obtained from feature knockout analysis for 34 miRNA signature are shown in Supplementary Table 1.

### Difference of expression profiles between early stage and advanced stage groups

We measured expression levels of the 10 highest ranked miRNAs in early stage and advanced stage groups. We observed a slight expression difference between early and advanced stage groups for 10 highest ranked miRNAs. Of the 10 highest ranked miRNAs, the mean expression values of hsa-miR-200c, hsa-miR-503, hsa-miR-1307, hsa-miR-361, hsa-miR-212, hsa-miR-592, hsa-miR-1185-1, hsa-miR-146b, hsa-miR-1468, and hsa-miR-769 are 13.34 ± 0.94, 3.44 ± 1.28, 10.16 ± 1.04, 8.35 ± 0.57, 2.20 ± 0.83, 1.93 ± 1.11, 0.24 ± 0.39, 9.03 ± 0.94, 2.50 ± 1.10, and 4.88 ± 0.70, respectively, in the early stage group, and 13.28 ± 0.77, 3.80 ± 1.39, 9.93 ± 1.12, 8.30 ± 0.55, 2.20 ± 0.80, 1.80 ± 1.11, 0.35 ± 0.39, 9.20 ± 0.96, 2.45 ± 1.06, and 4.75 ± 0.77, respectively, in the advanced stage group. Box-plot representation of expression difference in the early stage and advanced stage groups is given for the signature of 34 miRNAs in Supplementary Fig. 1.

#### KEGG pathway enrichment analysis

**Figure 3:**
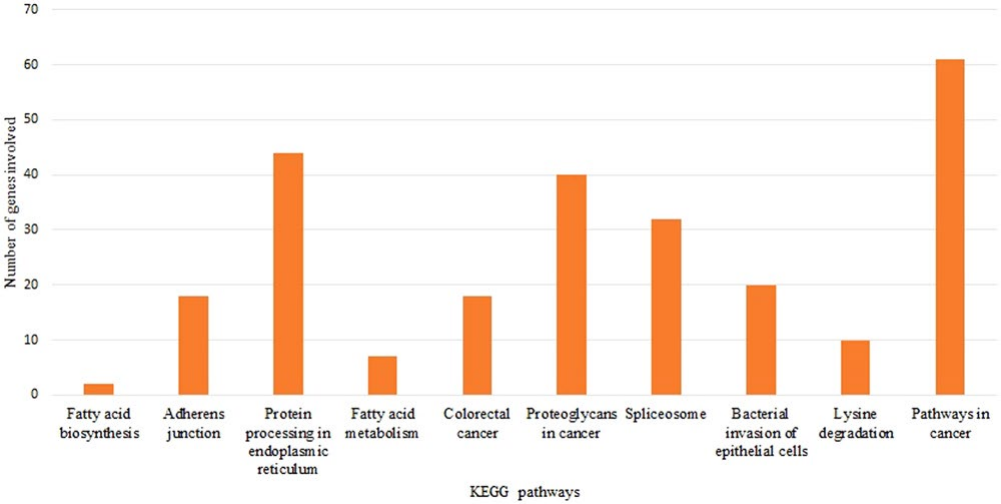
KEGG pathway analysis of the 10 highest ranked miRNAs.

To investigate the functional mechanism of the 10 highest ranked miRNAs, we employed KEGG pathway analysis using the DIANA-mirPath v.3 web server^69^. The 10 highest ranked miRNAs are significantly enriched in pathways involving fatty acid biosynthesis, fatty acid metabolism, adherens junction, protein processing in endoplasmic reticulum, cytokine-cytokine interaction, bacterial invasion of epithelial cells, spliceosome, and proteoglycans in cancer. The significantly enriched in KEGG pathways for the 10 highest ranked miRNAs and the target genes involved in each pathway are listed in Table 3. The 10 highest ranked miRNAs and the number of targeted genes are shown in Fig. 3. A detailed summary of the 10 highest ranked miRNAs, the enriched KEGG pathways and the number of targeted genes is provided in Supplementary Table 2.

**Table 3:** Enriched KEGG pathways and the corresponding target genes for the 10 highest ranked miRNAs.

KEGG pathway	p-value	Target genes
Fatty acid biosynthesis (hsa00061)	<1e-325	FASN
Fatty acid metabolism (hsa01212)	<1e-325	FASN TECR ACOX1
Adherens junction (hsa04520)	4.47E-06	TGFBR1, MET, WASL, SMAD2, ACTG1, IQGAP1, IGF1R, VCL, RHOA, TJP1, MLLT4, CDH1, CTNNB1, CTNNA1, WASF2, ACTN4, CREBBP
Protein processing in endoplasmic reticulum (hsa04141)	0.00083483	HSPA1A, EIF2AK1, SSR1, RAD23B, AMFR, UGGT1, YOD1, SEL1L, HSP90AA1, DNAJC10, UBE2E2, STT3B, HSPH1, PDIA6, RAD23A, PRKCSH, VCP, HSPA8, LMAN1, RPN2, DERL1, HSPA1B
Cytokine-cytokine receptor interaction (hsa04060)	0.002767508	IL6ST
Bacterial invasion of epithelial cells (hsa05100)	0.01255968	ARPC5L, MET, ITGB1, WASL, SEPT11, ACTG1, VCL, RHOA, CD2AP, CDH1, CLTA, WASF2, FN1, ARPC2
Spliceosome (hsa03040)	0.02884541	RBM25, HSPA1A, HNRNPA1, DDX23, PPIL1, U2SURP, PRPF8, SRSF1, HNRNPM, DHX15, HSPA8, DHX16, SRSF3, HSPA1B, SNRPC, SNRNP200, SRSF8
Proteoglycans in cancer (hsa05205)	0.03666157	PDCD4, MET, ITGB1, EZR, ARHGEF12, ACTG1, FRS2, IQGAP1, RHOA, ERBB3, ITGAV, LUM, HOXD10, FN1, MAP2K1, SDC4, TWIST1, VEGFA, MDM2, SMAD2, WNT5A, PPP1CC, ACTG1, TIAM1, IGF1R, AKT2, PTK2, CTNNB1, ITGA2, DDX5, GAB1

Most of the 34 miRNAs are prevalently involved in the biological pathways. For example, 30 miRNAs of the signature are significantly involved in the RAS signalling pathway, cGMP-signaling pathway, and cancer pathways by targeting 123, 90 and 229 genes, respectively. There are 29 miRNAs significantly involved in focal adhesion, PI3K-Akt signaling pathway, MAPK signaling pathway, and viral carcinogenesis. There are 28 miRNAs in proteoglycans in cancer pathway, ErbB signaling pathway, cAMP signaling pathway, and estrogen signaling pathway to name a few. Details of the miRNA signature involved in biological pathways and their targeted genes are listed in Supplementary Table 3.

#### Gene ontology analysis

**Figure 4:**
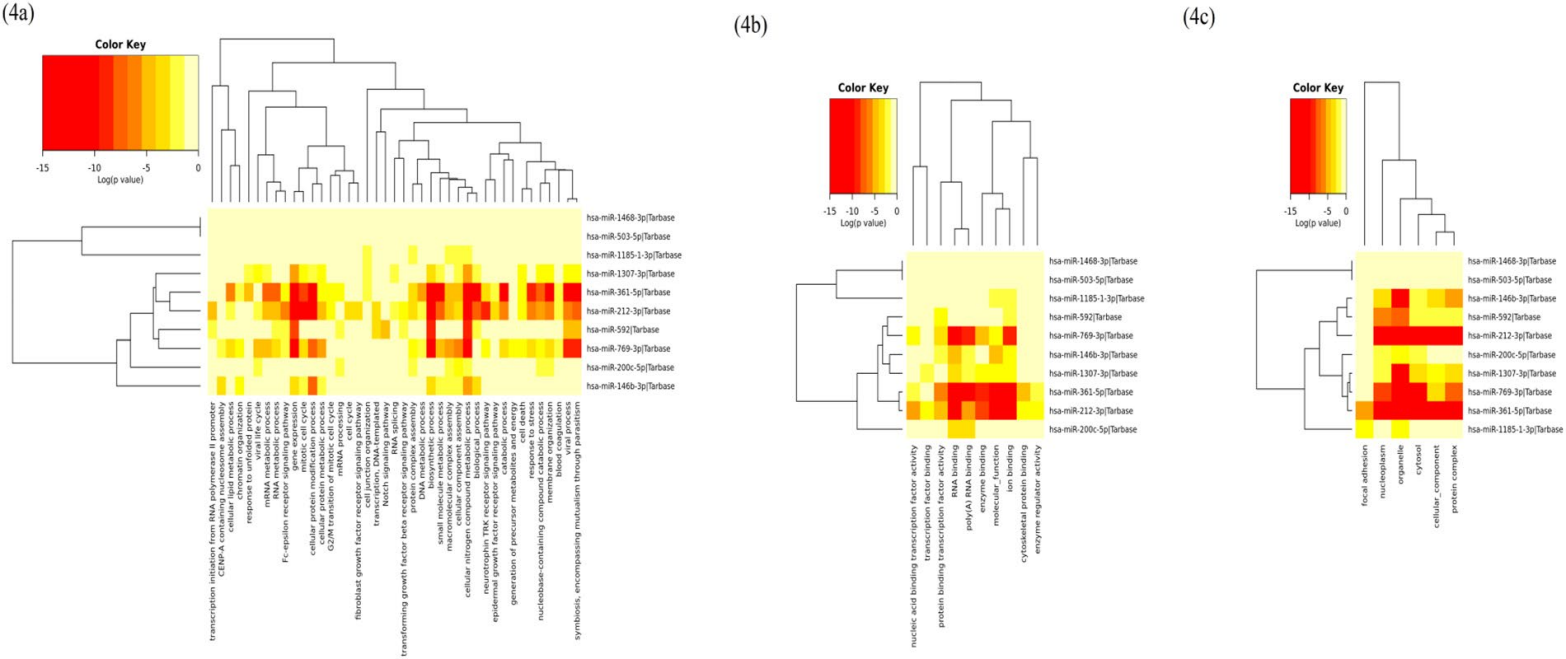
Gene ontology (GO) annotations for the 10 highest ranked miRNAs. GO enrichment analysis was performed for the 10 highest ranked miRNAs at three levels: biological process (**a**), molecular functions (**b**), and cellular component (**c**).

The biological significance of the 10 highest ranked miRNAs was analysed using GO annotations at three levels, includes biological process, molecular functions and cellular component. The 10 highest ranked miRNAs were highly enriched in five biological processes: mitotic cell cycle, cellular protein modification process, viral process, small molecule metabolic process, and symbiosis, encompassing mutualism through parasitism. The 10 highest ranked miRNAs were highly enriched in the molecular functions enzyme binding, RNA binding, and poly(A) RNA binding; the significantly enriched cellular components include protein complex, nucleoplasm, cytosol, organelle and focal adhesion. The enriched biological processes, molecular function and cellular components of the 10 highest ranked miRNAs are shown in Fig. 4(a–c). GO analysis of the 10 highest ranked miRNAs and the targeted genes for biological process, molecular function and cellular component are listed in Supplementary Tables 4, 5 and 6 respectively.

#### Survival analysis of the top ranked miRNAs

**Figure 5:**
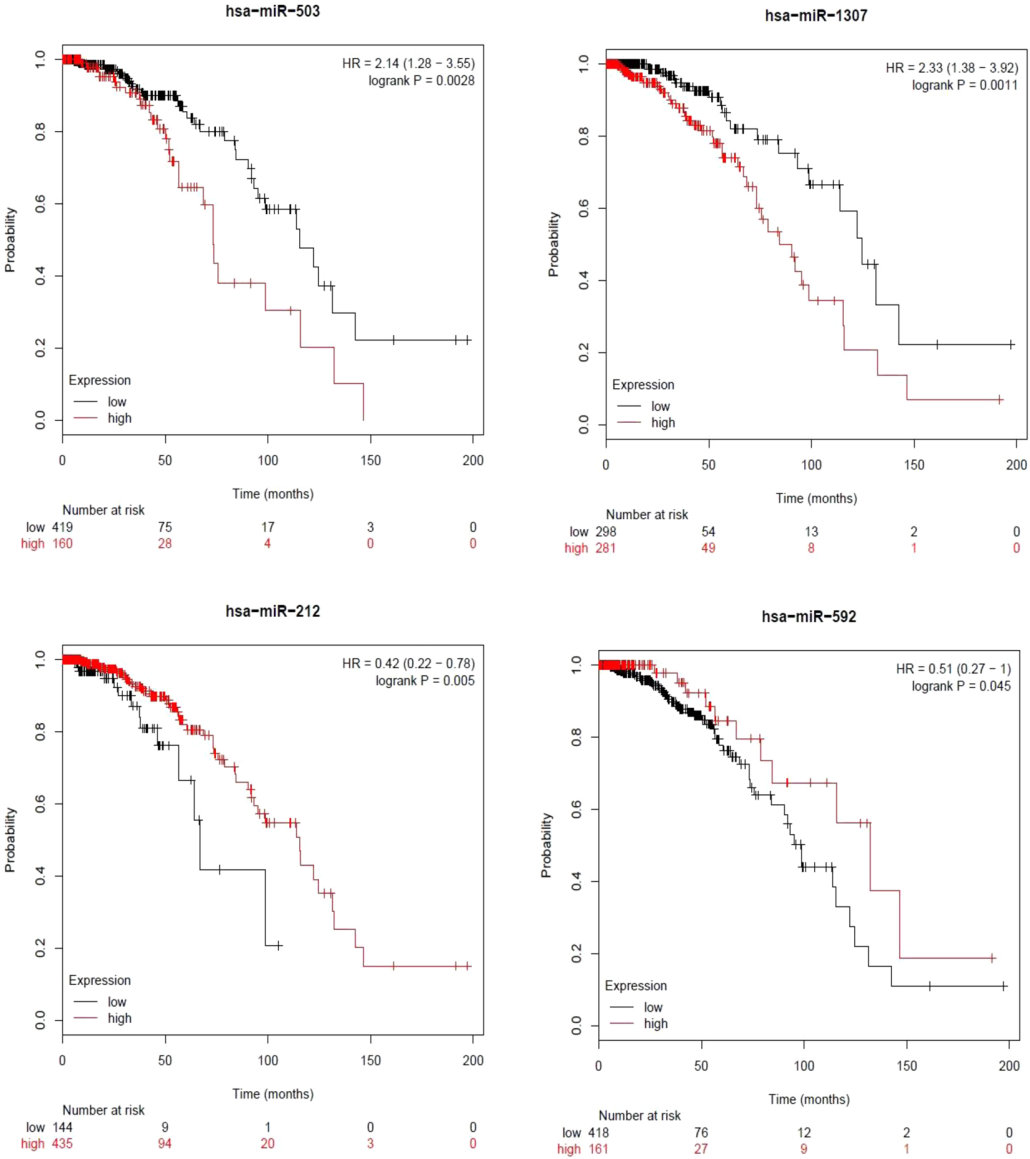
Kaplan-Meier plots of hsa-miR-503, hsa-miR-1307, hsa-miR-212, and hsa-miR-592 for the systemically treated breast cancer cohort.

Survival analysis was performed using Kaplan-Meier plotter^70^ to validate the prognostic value of the top ranked miRNAs. We selected the TCGA dataset and systematically evaluated the patient data using the Kaplan-Meier survival analysis. Four of the 10 highest miRNAs, hsa-miR-503, hsa-miR-1307, hsa-miR-212 and hsa-miR-592, were significantly associated with the prognosis of patients with breast cancer. These four miRNAs, hsa-miR-503, hsa-miR-1307, hsa-miR-212 and hsa-miR-592, obtained P-values of 0.0028, 0.0011, 0.005, and 0.045, respectively, and hazard ratios of 2.14, 2.33, 0.42 and 0.51, respectively, between the high and low expression groups. The Kaplan-Meier survival curves for the four miRNAs are shown in Fig. 5.

To confirm the association between the four miRNAs with overall survival, we utilized the METABRIC dataset. Two of the four miRNAs show significant association with prognosis in patients with breast cancer. Two miRNAs, hsa-miR-503 and hsa-miR-1307, obtained P-values of 0.046 and 0.0031, respectively, and hazard ratios of 0.82 and 1.37, respectively, between the high and low expression groups. Whereas another two miRNAs, hsa-miR-212 and hsa-miR-592, obtained P-values of 0.16 and 0.35, respectively, and hazard ratios of 0.87 and 0.9, respectively, between the high and low expression groups.

Another four of the 10 highest miRNAs, hsa-miR-200c, hsa-miR-1185, hsa-miR-146b and hsa-miR-769, were significantly associated with the prognosis of patients with breast cancer. These four miRNAs, hsa-miR-200c, hsa-miR-1185, hsa-miR-146b, and hsa-miR-769, obtained P-values of 0.00017, 1.4e-05, 0.0018, and 0.0078, respectively, and hazard ratios of 1.49, 0.6, 0.73, and 0.76, respectively, between the high and low expression groups. The Kaplan-Meier survival curves for the four miRNAs are shown in Supplementary Fig. 2.

Additionally, we estimated overall survival of the breast cancer patients using Multiple linear regression^71^, and observed that correlation between these four miRNAs and overall survival is better in the advanced stage group when compared to the early stage group. The correlation coefficient between actual and overall survival in early stage and advanced stage groups is 0.26 and 0.40, respectively. The correlation plots are shown in Supplementary Fig. 3.

## Conclusions

The challenges for early stage detection of breast cancer are that breast cancer is a heterogeneous disease with the potential for metastatic spreading at an early stage. Detecting cancer at a treatable stage and removing the lesions can prevent the development of lethal invasive cancers and would prevent death from breast cancer. Currently, it is widely reported that miRNAs can be potential biomarkers for various cancers. Identifying the disease-related miRNAs aids to improve the understanding of pathogenesis and diagnosis. Hence, various potential computational models have been developed to investigate the miRNA disease-association^9,72–74^. However, only a few studies focused on identifying a miRNA signature for the early stage detection of breast cancer. Accordingly, in this study, we proposed a novel miRNA-based classification method to categorize the early stage and the advanced stages of breast cancer. Recent development of personalized medicine and growing trend in applications of machine learning techniques improved the prognosis and cancer prediction. Various machine learning methods and feature selection algorithms have been widely used to identify the important factors that influence cancer progression, cancer recurrence, and cancer survival. Generally, machine learning based cancer prediction studies used mRNA/miRNA expression profiles, histological variables and clinical factors as input to the cancer prediction procedure^75–77^. Success in developing computational models for cancer predictions depends on understanding of biological knowledge and limitations of the training data set such as a small set of high-dimensional samples called “curse of dimensionality”^78^. However, the over-training problem can be coped with proper feature selection and cross-validation methods.

Hence, we proposed an SVM-based classifier called SVM-BRC that incorporated the feature selection method IBCGA to identify a miRNA signature that can distinguish early stage from advanced stage breast cancer. SVM-BRC identified a 34-miRNA signature and obtained a 10-CV accuracy, sensitivity, specificity, MCC and AUC of 83.16% 0.84, 0.81, 0.66 and 0.87, respectively. SVM-BRC obtained an average training accuracy of 80.38% ± 1.55%. Further, we ranked the identified miRNAs using MED scores. The significance of the 10 highest ranked miRNAs was validated using the literature. The importance of the top-10 miRNAs in breast cancer progression and other cancers is discussed. The prediction performance difference was measured for the 10 highest ranked miRNAs using feature knockout analysis. The functional mechanisms of the 10 highest ranked miRNAs were analysed using KEGG pathway enrichment and GO enrichment at three levels, including biological process, molecular functions, and cellular components. Survival analysis of the highest ranked miRNAs in the breast cancer cohort using the Kaplan-Meier curve revealed that four miRNAs, hsa-miR-503, hsa-miR-1307, hsa-miR-212, and hsa-miR-592, among the top-10 miRNAs were significantly (P ≤ 0.05) associated with the prognosis of breast cancer. We hope that our findings will help to improve the early stage detection methodologies by using the miRNA signature as a biomarker of breast cancer.

## Materials and Methods

### Dataset

The miRNA expression profiles of breast cancer cohort obtained from the Illumina HiSeq 2000 miRNA sequencing platform were obtained from TCGA database. We considered only the patients who underwent radiotherapy or targeted molecular therapy. Further, we divided the patients into early stage and advanced stage based on their pathological condition. After the filtering, the final balanced dataset contained 386 patients, with 193 patients in the early stage group and 193 in the advanced stage group, along with 503 miRNA expression profiles.

### SVM-BRC

Support vector machines (SVMs) are based on statistical learning theory^79^. The main idea of an SVM is to find the optimal hyperplane between the two classes. SVMs have been used to solve biological problems due to their potential discriminating ability. SVMs have been widely used to detect tumour markers^80^ and to perform cancer predictions^81^. Thus, we proposed an SVM-based classifier SVM-BRC including the feature selection method IBCGA to categorize early stage and advanced stage groups with breast cancers. The general formulation of the SVM is

1 $$Minimize\,\frac{1}{2}{\parallel w\parallel }^{2}+C\sum _{i=1}^{n}{S}_{i}$$

where *w* is vector of the hyperplane, *C* is the classifier parameter, *S*_*i*_ are the variables and *n* = number of vectors in the training dataset.

### Inheritable bi-objective combinatorial genetic algorithm (IBCGA)

To select a small set of miRNAs (signature) from a large number of expression profiles (503 miRNAs) we used a genetic algorithm (GA) based feature selection algorithm IBCGA^29^. The feature selection algorithm IBCGA uses an intelligent evolutionary algorithm^82^ to solve the large parameter optimization problem. IBCGA has been successfully applied in several bioinformatics problems, including the prediction of human ubiquitination sites^83^, the prediction of the regulatory roles of cyclic AMP receptor proteins^84^ and the estimation of survival time for cancer patients^85,86^.

In this study, we used IBCGA and identified a miRNA signature (m = 34 miRNAs) from a large number of miRNA expression profiles (n = 503 miRNAs) to distinguish the early stage and advanced stage groups with breast cancer. We used traditional terms of GA, GA-gene and GA-chromosome. The GA-chromosome of IBCGA consists of *n* binary GA-genes for feature selection and two 4-bit GA-genes for encoding parameters *C* and γ of SVM. Normalized miRNA expressions of patients with *n* miRNAs were used as input of IBCGA in designing the SVM-based classifier. The parameter setting of IBCGA was as follows: *r*_*start*_ = 10, *r*_*end*_ = 50, *N*_*pop*_ = 50, *G*_*max*_ = 60, and r = *r*_*start*_. We used the LibSVM package^87^ to implement SVM-BRC. The steps of IBCGA are as follows.

Step 1: (Initialization) Randomly generate a population of *N*_*pop*_ individuals.

Step 2: (Evaluation) Evaluate the fitness value of all individuals using the fitness function that is the prediction accuracy in terms of 10-fold cross-validation (10-CV).

Step 3: (Selection) Use a tournament selection method that selects the winner from two randomly selected individuals to generate a mating pool.

Step 4: (Crossover) Select two parents from the mating pool to perform orthogonal array crossover operation.

Step 5: (Mutation) Apply a conventional mutation operator to the randomly selected individuals in the new population. Mutation is not applied to the best individuals to prevent the best fitness value from deterioration.

Step 6: (Termination test) If the stopping condition for obtaining the solution is satisfied, output the best individual as the solution. Otherwise, go to Step 3.

Step 7: (Inheritance) If r < *r*_*end*_, randomly change one bit in the binary GA-genes for each individual from 0 to 1; increase the number r by one, and go to Step 3. Otherwise, stop the algorithm.

Step 8: (Output) Obtain a set of *m* miRNAs from the GA-chromosome of the best individual.

### Weka classifier

Weka has implementations of all major learning techniques for classification and regression methods. Some methods of Weka data mining software^88^ were used to compare SVM-BRC such as Random forest (RF), Multilayer perceptron (MLP), Sequential minimal optimization (SMO), Naïve Bayes, and Decision tree for classification to discriminate early stage and advanced stage groups with breast cancer.

We evaluated the prediction performance of SVM-BRC using the prediction accuracy (*ACC*), sensitivity (*Sn*), specificity (*Sp*), Matthews correlation coefficient (*MCC*), and area under the ROC curve (AUC).

2 $$ACC=\frac{TP+TN}{TP+TN+FP+FN}$$

3 $$Sensitivity=\frac{TP}{TP+FN}\,$$

4 $$Specificity=\frac{TN}{TN+FP}$$

5 $$MCC=\frac{TP\times TN-FP\times FN}{\sqrt{(TP+FP)(TP+FN)(TN+FP)(TN+FN)}}$$

where *TP* is true positive; *TN* is true negative; *FP* is false positive; and *FN* is false negative.

### KEGG and GO term enrichment analysis

We used DIANA-mirPath v3.0 for KEGG pathway analysis. Fisher’s exact t-test was used for enrichment analysis^89^. GO term analysis was employed to determine the involvement of the 10 highest ranked miRNAs in biological process, molecular functions and cellular components using mirPath v3.0. The DIANA-Tarbase algorithm in the mirPath web server was used to predict the experimentally validated miRNA targets^89^.

### Kaplan-Meier survival analysis

To identify the miRNAs associated with the prognosis of breast cancer patients, we employed Kaplan-Meier survival analysis using the mirPower-Kaplan-Meier plotter web-tool^70^. We selected TCGA breast cancer dataset, and the analysis was restricted to only patients systemically treated with chemotherapy.

## Electronic supplementary material

Supplementary Information

## Data Availability

All the data used in this analysis can be found at TCGA data portal.
