# Supplementary material for: Identifying a miRNA signature for predicting the stage of breast cancer
Source: Sci Rep. 2018 Oct 31;8:16138. doi: 10.1038/s41598-018-34604-3 (PMC6208346; doi:10.1038/s41598-018-34604-3)
Supplement: Supplementary file 1 — Supplementary Information [file 41598_2018_34604_MOESM1_ESM.pdf]

Identifying a miRNA signature for predicting the stage of breast cancer

Srinivasulu Yerukala Sathipati[1] and Shinn-Ying Ho[1, 2, 3*]

[1]Institute of Bioinformatics and Systems Biology, National Chiao Tung University, Hsinchu, Taiwan

[2]Department of Biological Science and Technology, National Chiao Tung University, Hsinchu, Taiwan

[3]Center For Intelligent Drug Systems and Smart Bio-devices (IDS2B), National Chiao Tung University, Hsinchu, Taiwan

*Corresponding author

Email address:

SYH: syho@mail.nctu.edu.tw

Supplementary Figures

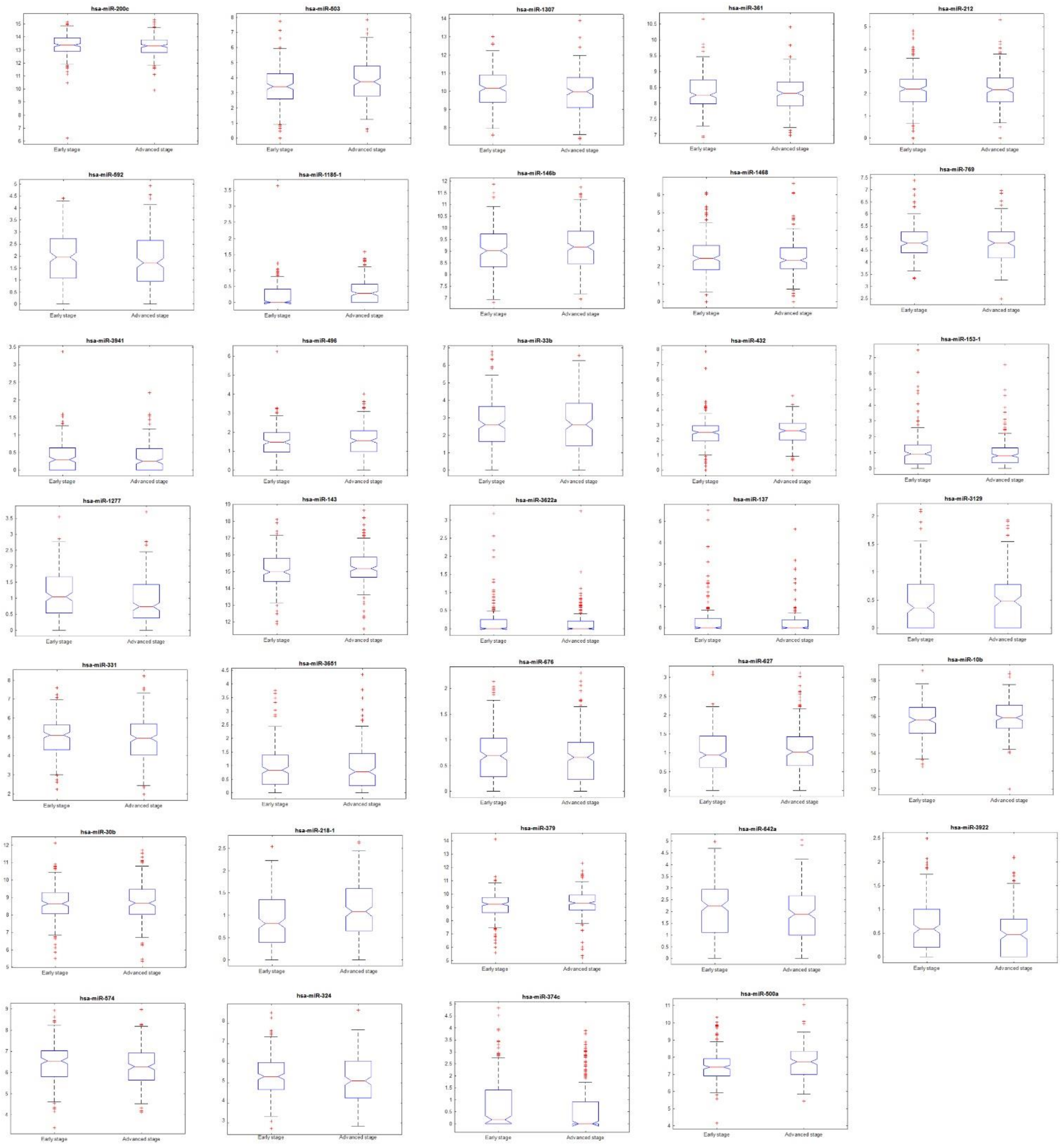

Supplementary Figure 1. Box-plot representation of expression difference between early stage and advanced stage groups.

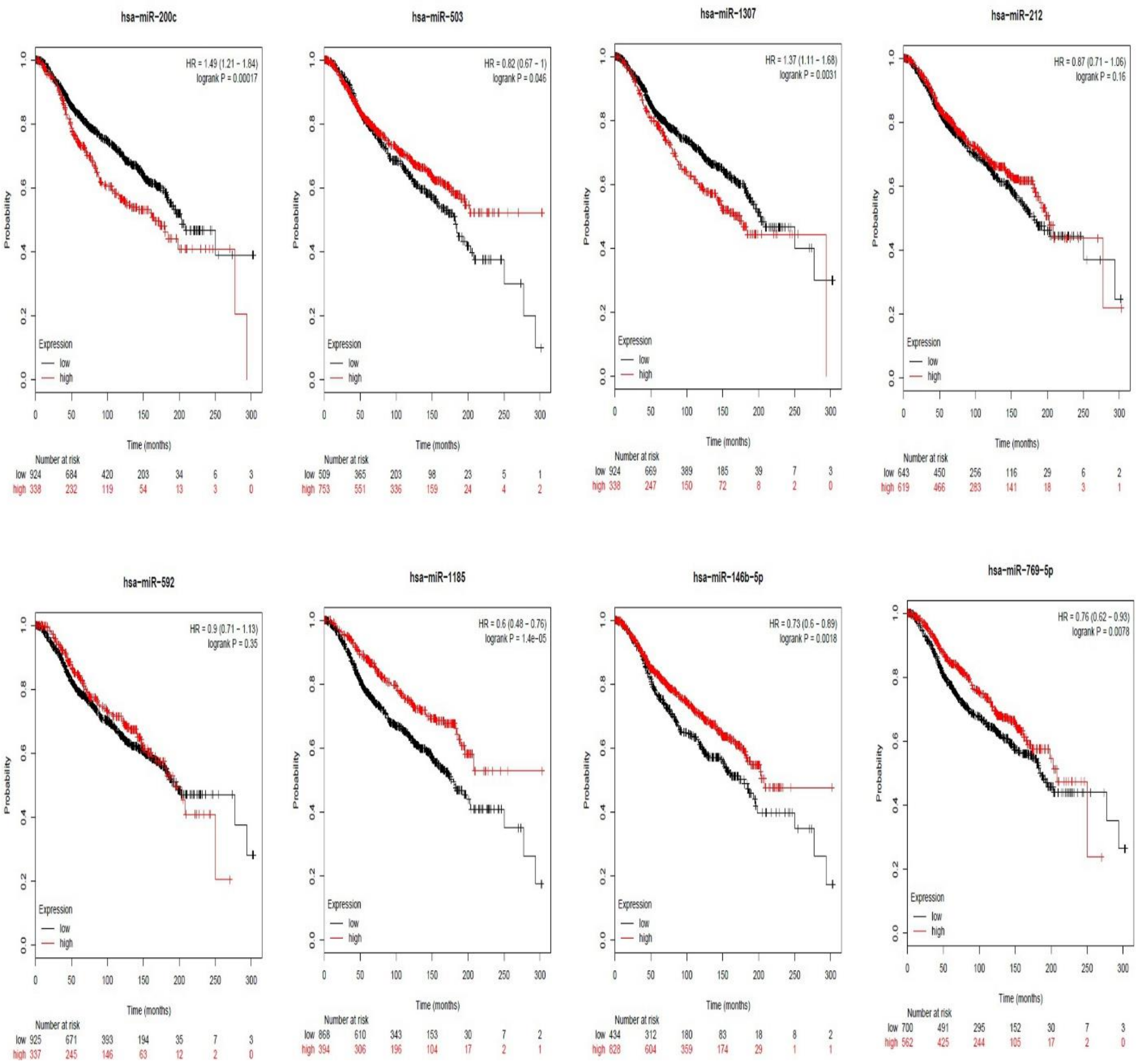

Supplementary Figure 2. Kaplan-Meier plots of eight highest ranked miRNAs for the systemically treated breast cancer cohort using the METABRIC dataset.

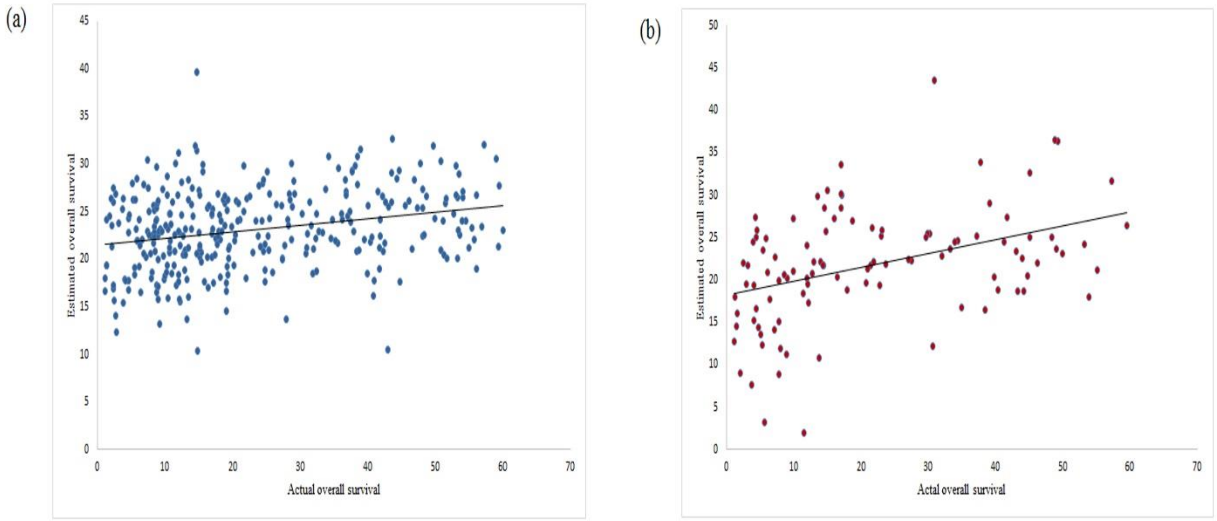

Supplementary Figure 3. Correlation plots for (a) early stage and (b) advanced stage groups. Actual overall survival is on X-axis and estimated overall survival is on Y-axis.

Supplementary Tables

Supplementary Table 1. MiRNA ranking using MED scores and feature knockout analysis for 34 miRNAs.

Rank	MiRNA	MED score	Accuracy difference (%)
1	hsa-miR-200c	69.6891	20.99
2	hsa-miR-503	65.02589	20.73
3	hsa-miR-1307	48.44561	21.25
4	hsa-miR-361	47.92746	21.25
5	hsa-miR-212	46.89119	20.99
6	hsa-miR-592	46.89119	19.95
7	hsa-miR-1185.1	43.26424	20.72
8	hsa-miR-146b	43.26423	19.69
9	hsa-miR-1468	34.45598	21.25
10	hsa-miR-769	30.82902	20.47
11	hsa-miR-3941	30.31089	21.51
12	hsa-miR-496	25.64768	21.25
13	hsa-miR-33b	23.57513	21.51
14	hsa-miR-432	20.98445	22.03
15	hsa-miR-153.1	19.43005	19.43
16	hsa-miR-1277	16.32125	20.46
17	hsa-miR-143	12.69432	21.25
18	hsa-miR-3622a	12.69429	20.47
19	hsa-miR-137	11.1399	21.24
20	hsa-miR-3129	11.13989	20.99
21	hsa-miR-331	8.031063	20.21
22	hsa-miR-3651	6.476673	20.47
23	hsa-miR-676	5.440403	21.25
24	hsa-miR-627	4.40414	20.73
25	hsa-miR-10b	-3.88601	20.98
26	hsa-miR-30b	-8.03106	20.98
27	hsa-miR-218.1	-8.03108	19.95
28	hsa-miR-379	-12.6943	21.51
29	hsa-miR-642a	-17.3575	20.47
30	hsa-miR-3922	-17.8757	20.47
31	hsa-miR-574	-30.3109	21.25
32	hsa-miR-324	-31.3471	21.51
33	hsa-miR-374c	-32.9016	20.21
34	hsa-miR-500a	-82.1244	22.28

Supplementary Table 2. Ten highest ranked miRNAs: enriched KEGG pathways.

KEGG pathway	p-value	Genes

Fatty acid biosynthesis	<1e-325	1
Fatty acid metabolism	<1e-325	3
Adherens junction	4.471E-06	17
Protein processing in endoplasmic reticulum	0.0008348	22
Cytokine-cytokine receptor interaction	0.0027675	1
Bacterial invasion of epithelial cells	0.0125597	14
Spliceosome	0.0288454	19
Proteoglycans in cancer	0.0366616	30
Lysine degradation	0.0960024	6
Biosynthesis of unsaturated fatty acids	0.1327072	1
Regulation of actin cytoskeleton	0.1723914	20
Colorectal cancer	0.2636787	3
TGF-beta signaling pathway	0.3314428	7
Signaling pathways regulating pluripotency of stem cells	0.4234695	10
Endocrine and other factor-regulated calcium reabsorption	0.5073056	3
Endocytosis	0.5451584	19
Pathways in cancer	0.5528573	29
Shigellosis	0.6166022	8
p53 signaling pathway	0.629832	10
MicroRNAs in cancer	0.652207	20
ECM-receptor interaction	0.683408	6
Cell cycle	0.7913113	12
Pyrimidine metabolism	0.8131436	3
Glioma	0.8151203	3
Epstein-Barr virus infection	0.8802326	13
Transcriptional misregulation in cancer	0.910793	15
Epithelial cell signaling in Helicobacter pylori infection	0.9118381	8
Cysteine and methionine metabolism	0.9707429	1
Oxidative phosphorylation	0.978327	10
Glycosaminoglycan degradation	0.9919812	2
SNARE interactions in vesicular transport	0.9975946	2

Supplementary Table 3. The miRNA signature is commonly involved in biological pathways. Significance between each miRNA and pathway is measured in p-value.

**KEGG pathway**	**No.of miRNAs involved**	**Target genes**	**p-value**
Proteoglycans in cancer	28	131	1.03338E-13

Hippo signaling pathway	25	98	2.13663E-09
TGF-beta signaling pathway	25	56	1.0579E-06
Axon guidance	25	83	1.0579E-06
ErbB signaling pathway	28	60	1.0579E-06
Glioma	25	44	2.62255E-06
Signaling pathways regulating pluripotency of stem cells	26	90	2.62255E-06
Prion diseases	18	17	9.55072E-06
Thyroid hormone signaling pathway	27	72	1.92126E-05
FoxO signaling pathway	25	85	2.01676E-05
Pathways in cancer	30	229	2.97297E-05
Morphine addiction	26	59	3.64208E-05
Adrenergic signaling in cardiomyocytes	28	83	6.81057E-05
Mucin type O-Glycan biosynthesis	21	17	7.46574E-05
Focal adhesion	29	123	0.000216734
GABAergic synapse	24	49	0.000284817
Long-term depression	26	40	0.000284817
PI3K-Akt signaling pathway	29	194	0.000284817
Renal cell carcinoma	23	45	0.000337557
Glycosaminoglycan biosynthesis - keratan sulfate	9	10	0.000626222
Wnt signaling pathway	25	82	0.000626222
Adherens junction	24	51	0.000829842
Prostate cancer	26	56	0.001305499
cAMP signaling pathway	28	115	0.001342091
Circadian rhythm	20	24	0.001434471
Transcriptional misregulation in cancer	27	102	0.001434471
Rap1 signaling pathway	28	119	0.001434471
Gap junction	26	54	0.001458716
Choline metabolism in cancer	25	65	0.002417336
Endocytosis	28	117	0.00347726
Amphetamine addiction	25	39	0.004088877
Melanogenesis	27	61	0.004570773
Chronic myeloid leukemia	23	45	0.004629624
Pancreatic cancer	25	43	0.004629624

Retrograde endocannabinoid signaling	26	61	0.004629624
Dopaminergic synapse	28	78	0.004629624
Ras signaling pathway	30	123	0.004629624
Tight junction	24	76	0.004925457
Endocrine and other factor-regulated calcium reabsorption	24	24	0.009556018
Glutamatergic synapse	25	62	0.009571108
mRNA surveillance pathway	23	53	0.011682309
Biotin metabolism	3	2	0.012715888
Glycosaminoglycan biosynthesis - heparan sulfate / heparin	12	14	0.012715888
Endometrial cancer	21	32	0.013348481
Sphingolipid signaling pathway	26	67	0.014036379
Melanoma	25	43	0.014555557
mTOR signaling pathway	23	38	0.014981044
Neurotrophin signaling pathway	26	69	0.016222327
Estrogen signaling pathway	28	57	0.016798557
ECM-receptor interaction	20	46	0.019853927
Acute myeloid leukemia	19	35	0.020859175
Long-term potentiation	24	41	0.02214123
Phosphatidylinositol signaling system	24	49	0.023848905
Colorectal cancer	24	38	0.023848905
Hepatitis C	26	75	0.023848905
cGMP-PKG signaling pathway	30	90	0.023848905
MAPK signaling pathway	29	133	0.023950862
Dorso-ventral axis formation	15	19	0.027386726
Lysine degradation	22	23	0.027450109
T cell receptor signaling pathway	24	60	0.027450109
Measles	25	72	0.027464695
Non-small cell lung cancer	24	34	0.027660066
Hedgehog signaling pathway	21	32	0.02918596
Ubiquitin mediated proteolysis	23	77	0.04169238
Insulin signaling pathway	27	77	0.04169238

Prolactin signaling pathway	24	40	0.042307821
Viral carcinogenesis	29	96	0.048261961

Supplementary Table 4. Ten highest ranked miRNAs signature: significantly enriched GO biological processes

GO Category	p-value	Genes
mitotic cell cycle	<1e-325	87
cellular protein modification process	<1e-325	329
viral process	<1e-325	108
small molecule metabolic process	<1e-325	287
symbiosis, encompassing mutualism through parasitism	<1e-325	119
membrane organization	<1e-325	113
biosynthetic process	<1e-325	528
gene expression	<1e-325	157
cellular nitrogen compound metabolic process	<1e-326	684
catabolic process	8.32667E-15	225
cellular component assembly	5.4845E-14	190
response to stress	6.42819E-14	242
macromolecular complex assembly	4.60354E-12	133
nucleobase-containing compound catabolic process	1.16445E-11	131
mRNA metabolic process	1.37178E-11	52
RNA metabolic process	5.91699E-11	54
neurotrophin TRK receptor signaling pathway	1.03916E-10	43
cellular protein metabolic process	4.97763E-09	73
cellular lipid metabolic process	3.64177E-07	30
Fc-epsilon receptor signaling pathway	4.46787E-07	29
protein complex assembly	5.3846E-07	101
DNA metabolic process	1.52289E-05	93
cell death	2.56391E-05	123
viral life cycle	5.18761E-05	24
transcription, DNA-templated	0.001133808	119
epidermal growth factor receptor signaling pathway	0.002227821	25
transcription initiation from RNA polymerase II promoter	0.004062725	21
G2/M transition of mitotic cell cycle	0.004378806	23

Supplementary Table 5. Ten highest ranked miRNA signature: significantly enriched GO molecular functions.

GO Category	p-value	Genes
enzyme binding	<1e-325	183
molecular_function	<1e-326	1638
RNA binding	<1e-327	315
poly(A) RNA binding	<1e-328	262
ion binding	<1e-329	690
protein binding transcription factor activity	1.031E-11	86
nucleic acid binding transcription factor activity	8.849E-06	101
cytoskeletal protein binding	0.0007619	68
enzyme regulator activity	0.0358469	67
transcription factor binding	0.0408334	42

Supplementary Table 6. Ten highest ranked miRNA signature: significantly enriched GO cellular components.

GO Category	p-value	Genes
cellular_component	<1e-325	1713
protein complex	<1e-325	517
nucleoplasm	<1e-325	227
cytosol	<1e-325	423
organelle	<1e-325	1299
focal adhesion	0.0001166	49
